# Natural selection *in utero* induced by mass layoffs: the hCG evidence

**DOI:** 10.1111/j.1752-4571.2012.00258.x

**Published:** 2012-12

**Authors:** Ralph Catalano, Claire Margerison-Zilko, Sidra Goldman-Mellor, Michelle Pearl, Elizabeth Anderson, Katherine Saxton, Tim Bruckner, Meenakshi Subbaraman, Julia Goodman, Mollie Epstein, Robert Currier, Martin Kharrazi

**Affiliations:** 1 School of Public Health, University of CaliforniaBerkeley, CA, USA; 2 Genetic Disease Screening Program, California Department of Public HealthRichmond, CA, USA; 3 Departments of Public Health and Planning, Policy and Design, University of CaliforniaIrvine, CA, USA

**Keywords:** human chorionic gonadotropin, mass layoffs, selection *in utero*

## Abstract

Evolutionary theory, when coupled with research from epidemiology, demography, and population endocrinology, suggests that contracting economies affect the fitness and health of human populations *via* natural selection *in utero*. We know, for example, that fetal death increases more among males than females when the economy unexpectedly contracts; that unexpected economic contraction predicts low secondary sex ratios; and that males from low sex ratio birth cohorts live, on average, longer than those from high sex ratio cohorts. We also know that low levels of human chorionic gonadotropin (i.e., hCG) measured in the serum of pregnant women predict fetal death. We do not, however, know whether male survivors of conception cohorts subjected to contracting economies exhibit, as theory predicts, higher hCG than those from other cohorts. We show, in 71 monthly conception cohorts including nearly two million California births, that they do. We thereby add to the literature suggesting that the economy, a phenomenon over which we collectively exercise at least some control, affects population health. Our findings imply that the effect arises *via* natural selection – a mechanism we largely ignore when attempting to explain, or alter, how collective choice affects our biology.

## Introduction

Natural selection has conserved mechanisms that spontaneously abort gestations that would otherwise yield relatively few grandchildren per unit of maternal investment (Trivers and Willard 1973; Stearns 1987; Forbes 1997; Møller 1997; Baird 2009). This selection *in utero* presumably contributes to the fitness of the population by keeping women from investing in less fit, while leaving them available to conceive fitter, offspring.

Selection *in utero* assumes that gestation somehow signals fetal fitness, that this signal must exceed some criterion to avoid spontaneous abortion, and that women autonomically raise that criterion when they encounter stressful environments that threaten their well-being and that of their less hardy children (Trivers and Willard 1973; Forbes 1997; Wells 2000; Catalano and Bruckner 2006). Selection *in utero* implies, therefore, that gestations just above the criterion in benign times would end in more stressful times.

Few historical data describe fitness of human gestations, but available records show that women who disproportionately bore sons and lived in areas with highly variable weather had fewer grandchildren than women who disproportionately bore daughters (Gabler and Voland 1994; Lummaa 2001). The relatively low fitness of sons in these stressful environments arose, primarily, from the high likelihood of death among young males. The death rate among male infants even now exceeds that of any other male or female age group under reproductive age and has done so in all societies and all years for which we have dependable data (Human Mortality Database 2010). This frailty among male infants persists despite the fact that mothers invest more energy in them than in daughters (Clutton-Brock 1991; Helle et al. 2002; Powe et al. 2010). The low return in grandchildren on maternal investment in sons would put gestations of males disproportionately near the criterion for spontaneous abortion.

Evidence of male preponderance among fetuses just above the criterion for spontaneous abortion includes that stressful times increase the ratio of male to female fetal deaths (Catalano et al. 2005). The ratio of male to female live births (i.e., the secondary sex ratio), moreover, reportedly falls in stressful times, with few exceptions (e.g., Stein et al. 2003), below that from cohorts in gestation during more benign times (Lyster 1974; Fukuda et al. 1998; Catalano 2003; Kemkes 2006; Catalano et al. 2008, 2009a; Saadat 2008; Helle et al. 2009). And males in low sex ratio birth cohorts exhibit lower infant mortality rates (Catalano et al. 2009b) and live longer (Catalano and Bruckner 2006; Bruckner and Catalano 2007, 2011) than those from other cohorts.

Further evidence of the near-criterion position of male fetuses comes from population endocrinology. As noted above, selection *in utero* requires that a gestation signal fitness to the mechanisms that spontaneously terminate pregnancy. Research suggests a complex, perhaps redundant, set of signals that vary over the course of gestation (Vigano et al. 2003; Erlebacher 2010; Sales et al. 2011). As noted by Haig (1993), Møller (1997), Forbes (2005), and Baird (2009), however, a relatively low level of gestational human chorionic gonadotropin (hCG) in maternal serum predicts spontaneous abortion better than other candidate signals (Dugoff et al. 2004; Goetzl et al. 2004; Cole 2010; Jelliffe-Pawlowski et al. 2010; Kirkegaard et al. 2011). Consistent with the assumption that male fetuses disproportionately rank low on fitness, gestations of males yield endemically lower hCG levels in maternal serum than those of females (Yaron et al. 2001; Cowans et al. 2009).

The robust correlation between low gestational hCG and spontaneous abortion has led to the suspicion that the hormone plays a role in sustaining pregnancies and may induce, rather than signal, fitness. Clinical trials have not supported this suspicion (Devaseelan et al. 2010).

The epidemiologic literature identifies at least one population stressor that intuition suggests should trigger selection *in utero* against gestations with low signals of fitness: mass layoffs (Catalano et al. 2010b). The U.S. Department of Labor defines mass layoff events as ‘fifty or more initial claims for unemployment insurance (UI) benefits filed against an employer during a 5-week period’ (Malley and Moutos 1996) and records UI claims resulting from these events as ‘mass layoff UI claims.’

Research reports elevated risk of stress-induced anxiety and subclinical depression among workers who lose their jobs (Dooley and Catalano 1980; Kessler et al. 1987; Conger et al. 1994; Catalano et al. 2010a). These losses appear to increase the risk of job losers and their families experiencing other adverse life events presumed to induce the stress response (Catalano et al. 1987; Rook et al. 1991; Vinokur et al. 1996). The widening ripples induced in the community by unexpected job losses include contagion-like increases in the stress of feared job loss (Leigh 1985; Liem and Liem 1988; Rook et al. 1991; Dua and Smyth 1993; Larson et al. 1994; Minchin 2009).

Consistent with the intuitive connection between the population stress induced by mass layoffs and selection *in utero*, the sex ratio of fetal deaths reportedly becomes more male biased, and the secondary sex ratio declines, when mass layoffs and joblessness increase above statistically expected levels (Catalano et al. 2005; Catalano et al. 2010b).

In sum, evolutionary theory, as well as empirical evidence from epidemiology, demography, and population endocrinology all lead to the suspicion that contracting economies affect the composition and fitness of contemporary human populations via selection *in utero* against males with low signals of fitness. We do not, however, know whether male survivors of gestational cohorts subjected to contracting economies exhibit higher hCG than those from other cohorts as we would expect if selection *in utero* terminated more low than high hCG gestations. We contribute to the literature by testing this expectation.

Our test requires that we specify which conception cohorts would most likely lose male fetuses when monthly mass layoffs exceed expected levels. The literature on fetal loss suggests that selection against less fit male fetuses may extend later in gestation than that against females. An estimated 25% of implanted (i.e., hCG-producing) gestations end within 6 weeks of implantation (Wilcox et al. 1999; Jukic et al. 2011). Female fetuses with chromosomal abnormalities predominate among these early abortuses (Evdokimova et al. 2000; Boklage 2005). At least 20% of the remaining pregnancies spontaneously abort even among young, healthy women in high-income societies with universal health care (Buss et al. 2006). Males predominate among these later losses (Evdokimova et al. 2000; Boklage 2005) in which chromosomal abnormalities appear less frequently than in the earlier losses.

Gestation, particularly of males, exhibits a critical window for selection between the 18th and 24th week when the risk of spontaneous abortion rises above the generally downward trend that begins after the 6th week of pregnancy (Goldhaber and Fireman 1991; Baird 2009). Consistent with selection *in utero*, males with low hCG and less robust reaction to exogenous stimuli predominate among these later losses. Clinical research reports that fetuses begin to exhibit the stress response to invasive therapeutic procedures at approximately the 18th week of gestation (Giannakoulopoulos et al. 1994, 1999; Marcus et al. 1999; Gitau et al. 2001; Myers et al. 2004). Basic research reports, moreover, that maturation of sensory mechanisms allows fetal cardiac response to, for example, sound in the mother’s environment around the 24th week of gestation (Lecanuet and Schaal 1996). Yet more research finds that motor activity appears greater among male than female fetuses but peaks for both in our critical period (i.e., late 2nd and early 3rd trimesters) and then decreases (Almli et al. 2001).

The literature described above suggests, and we test, the hypothesis that male infants in the 18th to 24th weeks of gestation when mass layoffs exceed expected values will exhibit levels of gestational hCG greater than expected from potentially confounding characteristics of their conception cohort, from levels among female infants in the same conception cohort, and from levels among male infants in earlier cohorts. The test uses data describing approximately 2 million infants conceived in California from May 2001 through March 2007.

## Materials and methods

### Data

#### hCG Data

All women in prenatal care by the 140th day of gestation have, by law, the opportunity to participate in California’s Genetic Disease Screening Program (GDSP). The program assesses the risk of chromosomal abnormalities using several blood analytes, including maternal serum hCG. GDSP contracts with regional laboratories to analyze blood samples from women who opt for testing. The laboratories follow a uniform assay protocol that uses an automated analytical system (Cunningham and Tompkinson 1999; Kazerouni et al. 2009).

We linked the hCG scores from the prenatal screening program with data from the California Department of Public Health birth registry for the years 2001–2007. The probabilistic linking procedure used combinations of mother’s/father’s/child’s names (first two letters, NYSIIS phonetic codes, and whole names), mother’s social security number, street address, phone numbers, residential ZIP code, mother’s birth date (year only and whole dates), date and time of birth, and birth facility.

For our dependent variable, we calculated median levels of maternal serum hCG (measured in international units per liter), assayed in the 14th through 21st weeks of gestation, among live born males from each of the 71 monthly cohorts conceived from May 2001 through March 2007. We used gestational age, derived primarily from ultrasound tests, to assign pregnancies to month of conception. If ultrasound results were not available, gestational age was estimated from the date of last menstrual period or physical exam (Dietz et al. 2007; Pearl et al. 2007). We included only singleton gestations in the study because conventions for assigning gestational hCG to survivors of multiple gestations have not been set, maternal serum screening of multiple gestations appears much less common than for singleton gestations (53% compared to 65%), and sex-specific selection *in utero* among twins appears different than that among singletons (Catalano et al. 2009a).

#### Mass Layoffs

We retrieved the number of UI claims resulting from mass layoffs in California for our test period from the US Department of Labor website (Unites States Department of Labor 2011). We assume, based on the literature (Cobb and Kasl 1977; Malley and Moutos 1996; Carroll 2003), that the *announcement* of intended mass layoffs gauges the degree to which the larger population perceives a threat to its economic security. Firms are legally required to notify workers of mass layoffs 60 days before the layoff occurs. We therefore consider mass layoff UI claims as indicators of stress on the population 2 months prior to the actual claims.

We needed 9 months of UI claims from mass layoffs for each conception cohort to estimate coefficients for exposure through 9 months of gestation. Our first cohort was conceived in May 2001 and our last in March 2007. We, therefore, retrieved mass layoffs for 79 months (i.e., from May 2001 through November 2007) to cover gestation of our 71 conception cohorts.

#### Covariates

Our test equation included nine covariates. First, we included the median female hCG score for each of the 71 cohorts. Including this covariate ensures that no measurement artifact (e.g., changes in assay kits) or any confounder that similarly affects both male and female gestations will induce inferential errors in test. Because hCG levels vary over gestation, we also included mean gestational age, in days, at time of maternal blood draw for each conception cohort. We included percentage of insulin-dependent mothers of male fetuses in each cohort because the literature reports correlations between maternal diabetes and hCG (Merviel et al. 2001). We, in addition, included mean maternal weight at time of the hCG test and mean maternal age at birth for mothers of males in each cohort. The test equation also included cohort percentage of mothers reporting Hispanic, non-Hispanic white, non-Hispanic African American, and Asian American race or ethnicity.

### Analyses

Consistent with the literature alluded to in the Introduction, we argue that cohorts of male infants exposed to greater-than-expected mass layoff announcements in approximately the 18th through 24th weeks of gestation will exhibit higher levels of gestational hCG than other cohorts. Because of the 2 month delay between mass layoff announcements and mass layoff UI claims, and because we cannot know when in a month conceptions began, or when layoffs were announced, we define exposed cohorts as those conceived 7 or 8 months before unexpectedly high levels of monthly mass layoff UI claims. We arrive at this window by adding 8.6 weeks (i.e., 60 days divided by 7) to the 18 weeks that start the critical period and the 24 weeks that end it. This leaves the span of 26.6–32.6 weeks. Dividing these by the 4.2 weeks in a typical month yields the window of 6.33–7.76 months. Our exposure period, therefore, starts approximately the second week of the 7th month of gestation and ends approximately in the last week of the 8th month.

Statistical tests of association essentially measure the degree to which two variables differ from their expected values in the same cases. The tests typically assume that the expected value of any observation is the mean of all observations. Variables measured over time, however, often violate this assumption because they exhibit ‘autocorrelation’ in the form of secular trends, cycles, or the tendency to remain elevated or depressed, or to oscillate, after high or low values. The expected value of an observation in such a series is not the mean of all observations but rather the value predicted by autocorrelation.

We used Box-Jenkins modeling to detect and model autocorrelation in our independent and, after adjusting for covariates, dependent variable (Box et al. 1994). More specifically, we tested our hypotheses through the following steps.

**1.** We used Box-Jenkins routines to decompose the number of mass layoff UI claims, measured in 1,000s, in California from March 2001 through December 2007 into statistically expected and residual (i.e., observed minus expected) values.

**2.** We regressed median male hCG scores among live births in each of the 71 conception cohorts on the nine covariates described above.

**3.** We applied Box-Jenkins routines to the residuals of the model estimated in Step 2 to detect autocorrelation. We added autoregressive and moving average parameters as needed to the model estimated in step 2 to ensure that the final residuals had a constant mean of 0, constant variance, and exhibited no autocorrelation.

**4.** We estimated the equation formed by adding, as a predictor variable, the residual values derived in Step 1 to the model developed in Steps 2 and 3. We aligned the data such that male cohort hCG was predicted from the UI claim residuals at the first through ninth month of gestation. Our theory predicts the coefficient for exposure at either or both the 7th or 8th month of gestation will be significantly (*P* < 0.05, 2-tailed test) < 0.

## Results

Approximately 65% of live births linked to prenatal screening records, yielding 2 057 433 singleton births. Table I compares live births from the screened gestations with all births in the test years. The groups appear similar although the screened group had a lower proportion of older women and a higher proportion of privately insured women. These differences probably result from older women being referred directly for amniocentesis rather than for the blood screening test, and uninsured women less frequently obtaining prenatal care and therefore less likely receiving any screening.

**Figure 1:**
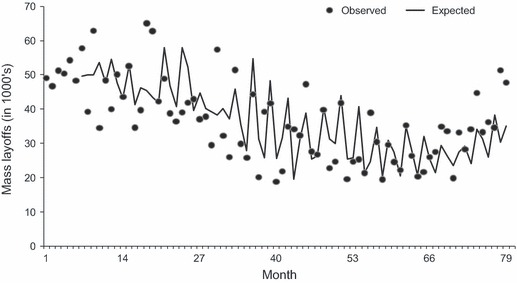
Expected and observed values of California mass layoff UI claims for 79 months starting May 2001 (first 6 months lost to modeling).

Step 1 above, using Box-Jenkins routines to decompose mass layoff UI claims into statistically expected and residual components, yielded the values plotted in Fig. 1. The following Box-Jenkins model best fits the UI series.


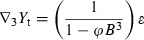


*Y*_t_ is the number of mass layoff UI claims in month t. ▽_3_is the difference operator indicating that *Y* at month t was subtracted from *Y* at month t + 3 to remove strong quarterly autocorrelation (i.e., high or low values at month t followed by similarly high or low values 3 months later). ϕ*B*^3^ is an autoregressive parameter indicating that a quarterly ‘memory’ remained in the series even after difference at 3 months. The estimated value of ϕ (i.e., .57; SE = 0.0998) suggested that this memory decreased geometrically by about 50% with the passage of each quarter.

**Figure 2:**
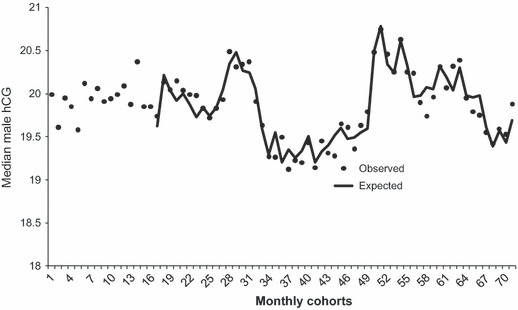
Observed and expected gestational hCG in 55 monthly cohorts of male infants conceived in California starting May 2001 (16 months lost to modeling).

Steps 2 and 3, regressing the median male hCG scores in the 71 conception cohorts on the nine covariates and adjusting residuals for autocorrelation, yielded the expected values shown in Fig. 2. The covariates model in Table 1 shows the coefficients estimated in this adjustment and their standard errors. Table 1 also shows that we detected and modeled autocorrelation in which high or low median levels of hCG among males repeated, adjusting for covariates, with similar, but smaller, high or low values 16 months later. This pattern implies that we could not predict hCG for cohorts conceived before September 2002. Our final test, therefore, used 55 cohorts conceived from September 2002 through March 2007.

**Table 1:** Comparison of study sample to all California births, 2002–2007

	Study sample (May 2001–March 2007)	California births 2002–2007
Singletons	2 057 443	3 203 026
% Male	51.1	51.2
Race/ethnicity, %		
White	26.8	29.0
African American	5.5	5.6
Asian	12.1	12.2
Hispanic	53.9	51.5
Maternal age (years), %		
<20	8.8	9.6
20–24	22.9	23.2
25–34	55.4	50.6
>34	12.9	16.7
Payment source (delivery), %		
Public	45.5	45.6
Private	51.4	48.7
Uninsured	1.1	2.3
Other/Unknown	2.0	3.3
Month prenatal care began, %		
1–2	72.1	66.9
3–4	23.5	23.9

**Figure 3:**
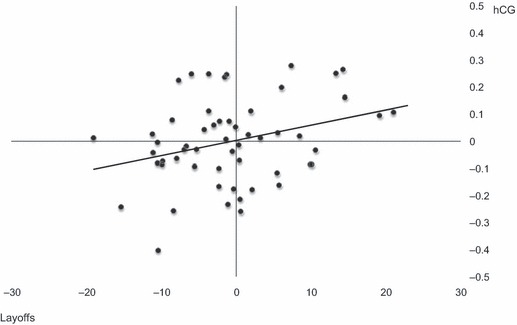
Scatter plot and regression line of adjusted median gestational hCG (IU/L) in monthly cohorts of male births over residuals of mass layoffs (1000s) in California for 55 months beginning September 2002.

Step 4, adding the unexpected mass layoff UI claims derived in step 1 to the model resulting from step 3, yielded the results shown in Table 2. Consistent with our argument, the cohorts of male infants conceived 8 months prior to unexpectedly high levels of claims exhibited higher median hCG levels than expected from history and from the specified covariates. Figure 3 shows a scatter plot and regression line for male cohort hCG, adjusted for covariates and autocorrelation, and unexpected mass layoff claims in the 8th month of gestation.

**Table 2:** Coefficients from covariates-only and full model predicting median gestational hCG among male infants for 71 monthly California conception cohorts starting May 2001

	Covariates model		Full model	
Variable	Coefficient	SE	Coefficient	SE
Median Female hCG	0.8063**	0.0529	0.7965**	0.0483
Mean age mothers of males	−0.0748	0.1848	−0.0795	0.1779
Mean weight mothers of males	−0.0773*	0.0387	−0.0863*	0.0383
Mean male gestational age	0.9884**	0.3447	1.0773**	0.3311
% white male	0.1079	0.5069	0.4665	0.4777
% African American male	10.3345	7.4615	14.9893	8.2895
% Hispanic male	−1.9895	1.1482	−2.2170	1.1667
% Asian male	−0.9091	4.8046	−1.7212	4.5951
% Insulin using mothers of males	68.4463*	29.9513	62.4579	33.9537
UI Claims in month 9 of gestation			0.0024	0.0036
UI Claims in month 8			0.0085*	0.0035
UI Claims in month 7			−0.0037	0.0040
UI Claims in month 6			−0.0030	0.0039
UI Claims in month 5			−0.0020	0.0041
UI Claims in month 4			−0.0058	0.0036
UI Claims in month 3			0.0027	0.0040
UI Claims in month 2			−0.0004	0.0036
UI Claims in month 1			0.0030	0.0039
Autoregression at 16th month	−0.5706**	0.1230	−0.6232**	0.1309

* *P* < 0.05; two-tailed test.

* *P < 0.01; two-tailed test.

We conducted several additional tests to estimate the robustness of our findings. First, we transformed the dependent variable to natural logarithms to determine whether variability in variation could have induced our results. The findings did not change. In a related test, we applied the routines that detect and control outliers in the dependent variable (Chang et al. 1988). We detected no outliers. We also used the routines that iteratively ‘pare’ statistically nonsignificant (*P* > 0.05; 2-tailed test) covariates from the final model (Liu and Hudak 1992). This routine left only median female hCG, mean maternal weight, and mean gestational age at blood draw among covariates in the final estimation but mass layoff UI claims remained significantly and positively related to median male hCG scores.

We also tested our hypothesis with three other configurations of our dependent variable. First, we estimated an equation with the male hCG deficit (i.e., median male hCG subtracted from median female hCG) as the dependent variable. This specification reflects the argument that the endemic difference in gestational hCG will shrink when stressors on the population induce selection against less fit fetuses. The coefficient for mass layoff UI claims remained significantly and, consistent with theory, inversely related to the males hCG deficit. Second, we used the ratio of male to female median hCG (i.e., the hCG sex ratio) as the dependent variable. The coefficient for mass layoff UI claims, consistent with the other results, again remained significantly >0. Third, we estimated an equation using only data for males (i.e., male median hCG as the dependent variable, female scores not among covariates). Because excluding median female hCG scores from the test leaves confounders shared by both sexes uncontrolled, we detected and controlled ‘level shifts,’ induced by changes over time in assay methods, in the residuals (Alwan and Roberts 1988). The results also supported our argument in that increasing mass layoff UI claims predicted higher median male hCG scores.

Our argument assumes that the previously reported association between labor market contraction and the secondary sex ratio would appear in our test population using mass layoffs, adjusted for autocorrelation, in the 8th month of gestation as the independent variable. To test this assumption, we followed the steps described above for our main test. We specified the number of males in each cohort as the dependent variable and included, as covariates, the number of females as well as three characteristics of the cohorts (i.e., mean maternal age, percent non-Hispanic white, and percent African American) that prior literature suggests could confound the association between the economy and the sex ratio. Results showed that, as expected from earlier research, the sex ratio of survivors of the conception cohorts declined as the number of mass layoffs increased above levels expected from autocorrelation.

While the results shown in Table 2 allow us to reject the null hypothesis, they convey little about the strength of association. We, therefore, made additional calculations to determine whether the discovered effect could actually change the ranking of conception cohorts on male hCG levels. First, we converted our continuous mass layoffs variable to a binary exposure. We followed the convention for creating such exposure variables by using a simple median split of months in which mass layoffs exceeded their expected values (i.e., had positively signed residuals from the Box-Jenkins model). We assigned a score of 1 to the 14 months with residual mass layoffs (in 1000s) above the median (i.e., 5.7909) and scored the remaining 57 months 0. Second, we estimated an equation with all the covariates shown in Table 1 and the binary exposure variable for the 8th month of gestation (i.e., mass layoffs announced in the 6th month of gestation). Results showed that male median hCG rose by .1985 IU/L in the exposed cohorts. Third, we estimated the implications of this finding for two intuitively informative unexposed conception cohorts. First, the lowest of the 71 median male hCG scores was 19.1200 IU/L and came, as expected, from an unexposed cohort. Our findings imply that exposing this cohort to stressful levels of mass layoff announcements would cull enough less fit male fetuses to raise its score to 19.3185 IU/L (i.e., 19.1200 + 0.1985). This increase means that the effect we estimated has sufficient strength to move the cohort from rank 71 (i.e., lowest) to 64th among all cohorts. Second, the cohort, also unexposed, at the median (i.e., 19.900) of all cohorts would rise from 36th to 19th (i.e., 20.0985) if exposed to these announcements.

## Discussion

We contribute to the literature by offering the first test of an important link in the suspected causal chain connecting population stressors to the secondary sex ratio. We find that male infants in the 18th to 24th weeks of gestation when mass layoffs exceed expected values exhibit levels of gestational hCG greater than expected from potentially confounding characteristics of their conception cohort, from levels among female infants in the same conception cohort, and from levels among male infants in earlier cohorts. This finding suggests, consistent with theory, both that the maternal stress response includes raising the level of fetal fitness needed to avoid spontaneous abortion and that hCG signals that fitness.

Contributions of this finding to basic science include mechanistic evidence of natural selection *in utero* responding to a stressor of contemporary human populations. Although earlier work offers indirect demographic and epidemiologic evidence of the effects of such selection, none provides evidence of suspected mechanisms responding to known populations stressors. We show that more-than-expected mass layoff announcements, predict, if not cause, a population array of hCG consistent with natural selection *in utero.*

Epidemiology has only recently acknowledged (e.g., Catalano and Bruckner 2005) the connection, long suspected by evolutionary theorists (e.g., Trivers and Willard 1973), between natural selection and fetal loss. Our work uses data and methods familiar to both groups to demonstrate that a known risk factor for fetal loss, low gestational hCG, may play an important role in natural selection. We further demonstrate that population stressors apparently sufficient to induce fetal loss and natural selection *in utero* include one already studied by epidemiologists – declining economies (Catalano et al. 2010a).

Our data do not allow us to determine whether, as implied by selection *in utero*, conception cohorts with higher levels of gestational hCG exhibit lower morbidity early in life than those with lower hCG. Linking pregnancy-screening data with infant health records would allow estimating the yield of cohort morbidity and mortality predictable from gestational hCG. These estimates might be useful for anticipating temporal variation in the need for preventive and treatment services in infant populations.

Our use of state-level data leaves unclear whether the labor markets that accounted for most of the mass layoffs also yielded most of the high gestational hCG cohorts. Using sub-state regions will require access to unpublished mass layoff data but would allow more inferential certainty.

We used median hCG scores in part because we wanted a conventional dependent variable for what readers might find an otherwise unconventional test. This choice, however, also made our test conservative in that a shifting criterion for spontaneous abortion affects only a small population of fetuses at the far left tail of the hCG distribution. Unrelated changes elsewhere in the distribution could, therefore, dilute the effect of selection *in utero* on median hCG scores. Future research should explore more sensitive measures of selection (e.g., the value defining the 10th percentile of hCG among males in each cohort) than cohort median hCG.

The strengths of our analyses include the number, size, and diversity of birth cohorts we analyzed. We know of no other data describing gestational hCG in as many as two million births from 71 monthly birth cohorts that include a broad array of racial and ethnic groups. This wealth of data allowed us to use time-series methods to detect selection *in utero* in the phenomenon to which it most logically applies – temporal variation in a signal of fitness among survivors of large, naturally occurring, conception cohorts exposed in gestation to varying doses of an ambient stressor.

Our findings add to the literature suggesting that the economy, a phenomenon over which we collectively exercise at least some control, affects human biology and population health (Catalano et al. 2010b). We contribute to the literature by showing that the effect arises, at least in part, from natural selection – a mechanism we largely ignore when attempting to explain, or alter, how collective choice affects our biology (Ness and Stearns 2008).

## Data archiving statement

Data deposited in the Dryad repository: doi:10.5061/dryad.h4j28d1n
